# Cudraflavone C Induces Tumor-Specific Apoptosis in Colorectal Cancer Cells through Inhibition of the Phosphoinositide 3-Kinase (PI3K)-AKT Pathway

**DOI:** 10.1371/journal.pone.0170551

**Published:** 2017-01-20

**Authors:** Hsien-Chuen Soo, Felicia Fei-Lei Chung, Kuan-Hon Lim, Veronica Alicia Yap, Tracey D. Bradshaw, Ling-Wei Hii, Si-Hoey Tan, Sze-Jia See, Yuen-Fen Tan, Chee-Onn Leong, Chun-Wai Mai

**Affiliations:** 1 School of Medicine, International Medical University, Bukit Jalil, Kuala Lumpur, Malaysia; 2 Center for Cancer and Stem Cell Research, International Medical University, Bukit Jalil, Kuala Lumpur, Malaysia; 3 School of Pharmacy, University of Nottingham Malaysia Campus, Jalan Broga, Semenyih, Selangor, Malaysia; 4 School of Pharmacy, Centre for Biomolecular Sciences, University of Nottingham, University Park, Nottingham, United Kingdom; 5 School of Postgraduate Studies, International Medical University, Bukit Jalil, Kuala Lumpur, Malaysia; 6 School of Pharmacy, International Medical University, Bukit Jalil, Kuala Lumpur, Malaysia; University of Navarra, SPAIN

## Abstract

Cudraflavone C (Cud C) is a naturally-occurring flavonol with reported anti-proliferative activities. However, the mechanisms by which Cud C induced cytotoxicity have yet to be fully elucidated. Here, we investigated the effects of Cud C on cell proliferation, caspase activation andapoptosis induction in colorectal cancer cells (CRC). We show that Cud C inhibits cell proliferation in KM12, Caco-2, HT29, HCC2998, HCT116 and SW48 CRC but not in the non-transformed colorectal epithelial cells, CCD CoN 841. Cud C induces tumor-selective apoptosis *via* mitochondrial depolarization and activation of the intrinsic caspase pathway. Gene expression profiling by microarray analyses revealed that tumor suppressor genes *EGR1*, *HUWE1* and *SMG1* were significantly up-regulated while oncogenes such as *MYB1*, *CCNB1* and *GPX2* were down-regulated following treatment with Cud C. Further analyses using Connectivity Map revealed that Cud C induced a gene signature highly similar to that of protein synthesis inhibitors and phosphoinositide 3-kinase (PI3K)-AKT inhibitors, suggesting that Cud C might inhibit PI3K-AKT signaling. A luminescent cell free PI3K lipid kinase assay revealed that Cud C significantly inhibited p110β/p85α PI3K activity, followed by p120γ, p110δ/p85α, and p110α/p85α PI3K activities. The inhibition by Cud C on p110β/p85α PI3K activity was comparable to LY-294002, a known PI3K inhibitor. Cud C also inhibited phosphorylation of AKT independent of NFκB activity in CRC cells, while ectopic expression of myristoylated AKT completely abrogated the anti-proliferative effects, and apoptosis induced by Cud C in CRC. These findings demonstrate that Cud C induces tumor-selective cytotoxicity by targeting the PI3K-AKT pathway. These findings provide novel insights into the mechanism of action of Cud C, and indicate that Cud C further development of Cud C derivatives as potential therapeutic agents is warranted.

## Introduction

Colorectal cancer (CRC) is the third most common type of cancer and is one of the leading causes of cancer-related mortality worldwide, resulting in approximately 700,000 deaths every year [1, 2]. Despite aggressive screening and public health promotion, the global burden of CRC is anticipated to rise by 60% by 2030 [3]. Furthermore, despite recent advancements in targeted therapeutics, the 5-year survival rates remain low, particularly in patients diagnosed with advanced disease [4]. Thus, discovery of novel chemotherapeutic agents is imperative.

In the recent years, large-scale profiling of the cancer genome has uncovered druggable oncogenic pathways critical for driving CRC [2, 5–7]. The most common of which include excessive PI3K-AKT signaling driven by insulin-like growth factor 2 (IGF2) overexpression, phosphatidylinositol-4,5-bisphosphate 3-kinase catalytic subunit alpha (PIK3CA) mutations and phosphatase and tensin homolog (PTEN) mutations and deletions. Combined, these alterations are found in approximately 40% of CRC [2]. The PI3K-AKT signaling pathway has recently emerged as a promising target for cancer therapy. PI3K is a tyrosine kinase that regulates numerous processes that are important for cell survival. Upon activation by receptor tyrosine kinases, growth factors receptors, integrins, cytokine, G-protein-coupled receptors, and other stimuli, PI3K phosphorylates phosphatidylinositol 4, 5-bisphosphate (PIP2) to phosphatidylinositol (3, 4, 5)-trisphosphate (PIP3). In turn, PIP3 activates PDK1 which phosphorylates AKT at Thr308, leading to partial activation of AKT. AKT is fully activated upon further phosphorylation at Ser473 by the mTOR complex 2 (mTORC2) [8, 9]. Activated AKT regulates cell growth through a multitude of downstream targets including the regulation of mTOR signaling, inhibition of pro-apoptotic proteins (e.g. BAD, CASP9 and FOXO), phosphorylation of the CDK inhibitors p21 and p27 and regulation of NFκB signaling by phosphorylating IKKα and MAP3K8 [10].

Numerous studies have indicated the potential of inhibiting PI3K-AKT signaling as a strategy for treating cancer. Indeed, several PI3K-AKT inhibitors such as buparlisib, duvelisib and taselisib are currently being tested in Phase II and III clinical trials against a variety of solid tumors as well as hematologic malignancies [11]. Of note, idelalisib (P110δ inhibitor) received FDA approval in July 2014 for the treatment of leukemia and indolent non-Hodgkin's lymphomas [11]. A recent study also showed that inhibition of the p110δ PI3K isoform in regulatory T cells triggers antitumor immune response, indicating an alternative pathway through which PI3K inhibitors could target cancers which are not directly driven by PI3K overactivation [12].

**Fig 1:**
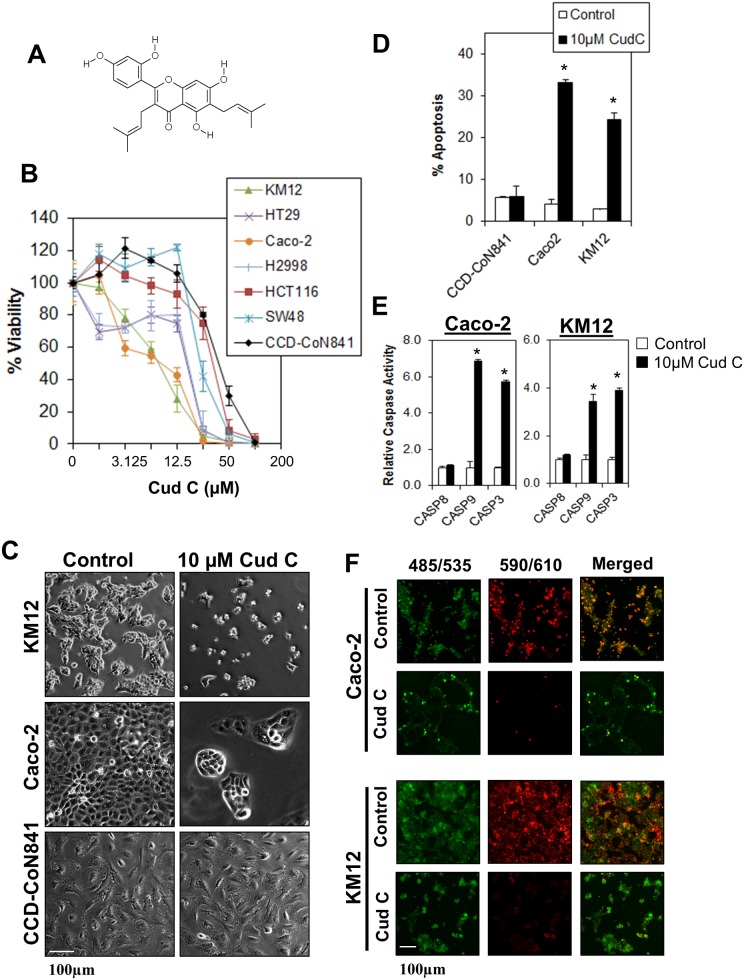
Cudraflavone C induces tumor-specific cell death in colorectal cancer cells. (A) Chemical structure of Cud C. (B) KM12, HT29, Caco-2, HCC2998, HCT116 and SW48 colorectal cancer cells were exposed to various concentrations of Cud C for 72 hours. Cell viability was recorded using CellTitre Glo^®^ luminescence assay. (C) KM12, Caco-2 and CCD 841 CoN were treated with 0.1% DMSO (control) or 10μM Cud C (Cud C) for 72 hours followed by microscopy analysis (×100 magnification). (D) Apoptotic cell death in KM12, Caco-2 and CCD 841 CoN cells was quantified using Annexin V/7-AAD flow cytometry at 72 hours following treatment. (E) Caspase activities in KM12 and Caco-2 cells were assessed by Caspase Glo^®^ assay at 72 hours following treatment. (F) 10μM Cud C induced mitochondrial membrane depolarization. Caco-2 and KM12 cells stained with JC-1 at 72 hours after treatment with Cud C. The green dye represents JC-1 monomers in cytoplasm while the red dye represents JC-1 aggregates in nucleus. Cells were observed under fluorescence microscope (×100 magnification). All data represent the mean ± s.d. from at least three independent experiments. Symbol “*” presents the statistical significance concluded from Student’s independent *t*-test with p-value ≤0.05.

In an attempt to identify potential therapeutics that are tumor specific, we conducted a high-throughput screen using a diverse chemical library and identified cudraflavone C (Cud C) as a tumor-specific agent against CRC cells (Fig 1A). Cud C is a flavonol which has been shown to inhibit melanin production *via* tyrosinase inhibition [13], inhibit pancreatic lipase [14], and inhibit activation of herpes simplex virus (HSV) [15]. Importantly, Cud C also exhibits anti-proliferative activities against human melanoma cells [16], hepatocellular carcinoma and gastric carcinoma cells [17]. It is noteworthy, however, that these studies did not encompass the pathway in which Cud C acted upon. A related compound, cudraflavone B, induces apoptosis in human oral cancer cells by modulating mitogen-activated protein kinase (MAPK), sirtuin-1 and Nuclear factor-κB (NFκB) pathways [18]. However, the molecular mechanism underlying the anti-proliferative effects of Cud C have yet to be fully elucidated.

In this study, we show that Cud C induces tumor-selective apoptosis in CRC cells by inducing the intrinsic apoptosis pathway and inhibition of PI3K-AKT signaling, a key signal transduction system which is frequently deregulated in human cancers.

## Materials and Methods

### Ethical approval

International Medical University Joint Committee on Research and Ethics (IMU-JC) approved the use of human cell lines in this study (BMS I01/2015). The research project did not involve any human, vertebrate animals, embryos or human biopsy tissues.

### Isolation and characterization of cudraflavone C

The bark of the hybrid species *Artocarpus heterophyllus x integer* (jackfruit), commonly cultivated in Malaysia and Indonesia, was collected in Malacca, Malaysia (2°13'45.08" N, 102°11'20.74" E). The plant was grown by a local family (private land), from whom permission was obtained for sufficient bark material to be collected. The plant, which was not an endangered or protected species, was identified by Dr. L.G. Saw (Forest Research Institute Malaysia) and the voucher specimens (herbarium no. KLU48746) were deposited at the Herbarium of the University of Malaya. The dried ground bark was initially defatted with hexanes (3 x 10 L) and subsequently extracted with ethylacetate, EtOAc (3 × 10 L) at room temperature for 72 hours. The EtOAc extracts were combined, and concentrated to afford 60 g of crude extract, which was then chromatographed over silica (column chromatography, 8 × 12 cm) eluted with EtOAc-hexanes (5:1), EtOAc, and EtOAc-MeOH (ethylacetate-methanol) (4:1) (affording step-wise increased polarity) to produce 12 combined fractions (F1–F12). Fraction F9 (4.5 g) was chromatographed further over silica (column chromatography, 4 × 12 cm), eluted with EtOAc and EtOAc-MeOH (4:1) (step-wise increase of polarity) to yield four flavonoid containing sub-fractions (F9-2 to F9-5), from which, F9-2 was purified by preparative centrifugal thin layer chromatography (silica), eluted with CHCl_3_-hexanes (4:1) and CHCl_3_-MeOH (4:1) (step-wise increase of polarity) to give 20 mg Cud C. Cud C is a light orange amorphous powder possessing the following properties; ultraviolet, UV (ethanol) λ_max_ (log ε) 210 (4.51), 232 (4.21); 261 (4.49), 314 (3.93) nm; infrared, IR (potassium bromide) *v*_max_ 3364, 1649 cm^-1^; high resolution electron spray ionization mass spectrometry, HRESIMS m/z 423.1811 (M^+^H)^+^ (calculated for C_25_H_26_O_6_ + H^+^, 423.1808); ^1^H and ^13^C nuclear magnetic resonance data, NMR (S1 Fig) consistent with those reported previously [19].

### Cell lines and cell culture

HT29, Caco-2, HCT116 and SW48 human CRC cell lines as well as non-transformed CCD 841 CoN human epithelial colon cells were purchased from the American Type Culture Collection, while National Cancer Institute, USA provided the KM-12 and HCC2998 cell lines. All CRC cells were maintained in RPMI 1640 medium while CCD 841 CoN cells were maintained in Dulbecco’s Modified Eagle Medium (DMEM). All media contained 10% fetal bovine serum (FBS) and 1% penicillin-streptomycin (Sigma-Aldrich, USA). Cells were cultured in a dedicated cell culture incubator at 37°C, 5% CO_2_ using standard cell culture methods optimized in previous studies [20, 21].

### Luminescent cell viability assay

Concentration-response curves and IC_50_ values were calculated using the Cell Titre-Glo^®^ Luminescent Cell Viability Assay kit (Promega, USA). Briefly, cells were plated in 96-well plates for 24 hours followed by Cud C treatment for 72 hours Luminescence was measured using SpectraMax M3 Multi-Mode Microplate Reader (Radnor, USA). The experiments were validated using CRC treated with 5-fluorouracil (Sigma-Aldrich, USA), the clinically used anti-cancer agent. Cell viability was expressed as a percentage of the vector-treated control.

### Detection of apoptosis by annexin V flow cytometry

Apoptotic events within a cell population were determined using the PE Annexin V Apoptosis Detection Kit (BD Biosciences, USA) at 72 hours following Cud C treatment as described previously [22–25].

### Caspase activation

The activity of caspase 3/7, 8 and 9 were measured at 72 hours after Cud C treatment using Caspase-Glo 3/7, Gaspase-Glo 8, and Caspase-Glo 9 Assay kits (Promega, Madison, WI, USA) according to the manufacturer’s instructions.

### Mitochondrial membrane depolarization assay

The effect of Cud C on mitochondrial membrane potential was assessed by JC-1 staining. Briefly, Caco2 and KM12 cells were seeded (1.5 x 10^5^ cells/mL) in a 6-well plate for 24 hours. The cells were treated with or without Cud C (1–100μM) for another 72 hours. Cells were then incubated with 5μg/mL of JC-1 dye (Merck, Darmstadt, Germany) for 30 minutes in dark at room temperature. Cells were subsequently washed with complete medium twice to remove excess dye. Fluorescence images were captured and processed using a Nikon Ti-U microscope.

### Microarray and connectivity map analysis

Caco-2 cells were treated with 10 μM Cud C or vehicle-only control (1% DMSO) for 48 hours. Total cellular RNA was isolated using the Qiagen RNA isolation kit (Qiagen, USA) according to the manufacturer’s protocol. The RNA samples were subjected to microarray analyses using a GeneChip^®^ Human Transcriptome Array 2.0 kit (Affymetrix, USA). Results were analyzed using Expression Console^™^ (Affymetrix, USA) and Affymetrix Transcriptome Analysis Console v3.0 (Affymetrix, USA). Genes which were up- or down-regulated ≥2-fold in treated cells compared to control cells were identified. The data were further analyzed by Connectivity Map analyses (CMap) [20, 26–28]. The results obtained from the CMap were further corroborated with target prediction results by SwissTargetPrediction (http://www.swisstargetprediction.ch/) [29, 30]. All microarray-related data are retrievable from the National Center for Biotechnology Information’s Gene Expression Omnibus (Accession Number: GSE78943).

### Quantitative real-time PCR analysis

cDNA was obtained from the High Capacity RNA-to-cDNA Master Mix (Applied Biosystems, USA) according to the manufacturer`s protocol. Gene expression levels were measured by quantitative real-time PCR (qPCR) using the FastStart Universal SYBR Green Master reagent (Roche, USA). The results were recorded by Bio-Rad iQ5 real-time PCR detector system (Bio-Rad, USA). Data analysis was performed using Bio-Rad iQ5 Optical System Software v1.0. We used forward and reverse primer sequences as described in S1 Table. All qPCR reactions were according to the following condition: 94°C (3 min) and then 94°C (40 seconds), 60°C (40 seconds), and 72°C (25 seconds) with a total of 40 cycles. The qPCR results of respective genes were normalized using GAPDH as the house keeping gene (20).

### Luminescent cell free PI3K lipid kinase assay

The effects of Cud C on PI3K p110α/p85α, p110β/p85α, p110δ/p85α, and p120γ were quantified using PI3K-Glo^™^ Class I Profiling Kit (Promega, Madison, WI, USA) according to the manufacturer’s instructions. Cud C (100 μM) was incubated with respective PI3K isoforms. Detection reagent was introduced before luminescence was recorded using SpectraMax M3 Multi-Mode Microplate Reader (Radnor, USA). The assay was validated using a known PI3K inhibitor, LY-294002 (Sigma-Aldrich, USA).

### Transfection

Transient transfection of plasmids into cells was performed using X-treme GENE HP DNA transfection reagent (Roche Diagnostics, IN, USA) according to on the manufacturer’s instructions. Constitutively active myristoylated AKT (Addgene plasmid # 9008) was a gift from William Sellers [31].

### Protein isolation and immunoblotting

Ice-cold lysis buffer consisting of 1% NP-40, 1 mM DTT and protease inhibitors in PBS were used to extract protein. A total 50 μg protein was subjected to sodium dodecyl sulfate polyacrylamide gel electrophoresis (SDS-PAGE) followed by immunoblotting as described in previous studies [22, 32–33]. Primary monoclonal antibodies that target phosphorylated AKT (p-AKT) S473 and T308, as well as total AKT were obtained from Cell Signaling, USA. A mouse monoclonal antibody against GAPDH was obtained from Santa Cruz Biotechnology. The results were viewed using ChemiDoc^™^ XRS+ molecular imager (Bio-Rad Laboratories, USA).

### Statistical analysis

All results were presented as mean ± standard deviation (s.d.) from at least three independent experiments. Statistical significance was determined by Student’s independent *t*-test through SPSS (version 18.0) for Windows. Test results were regarded as statistically significant when the *p*-value was not more than 0.05 unless otherwise specified.

## Results

### Selective anti-tumor effect of Cud C against CRC cells

To determine the anti-tumor effects of Cud C (Fig 1A), CRC cells (KM12, HT29, Caco-2, HCC2998, HCT116 and SW48) were treated with Cud C for 72 hours, and cell viability recorded using the CellTiter-Glo^®^ assay. Cud C elicited dose-dependent anti-proliferative activity against all CRC cells tested with IC_50_ values in the micromolar range (Fig 1B and Table 1). We also compared the results with IC_50_ of 5-fluorouracil in the same cell lines (Table 1). Cud C induced significant morphological changes in KM12 and Caco-2 but not CCD 841 CoN cells (Fig 1C). Further analyses revealed that Cud C induced significant apoptosis in Caco-2 and KM12 CRC cells but not in the non-transformed CCD 841 CoN cells (Fig 1D). Treatment with Cud C also resulted in a significant increase in caspase 3/7 and 9 activities, without eliciting a significant raise in caspase 8 activity (Fig 1E). Consistently, 10μM Cud C induced mitochondrial depolarization in Caco-2 and KM12 cells (Fig 1F). These results suggest that Cud C induces significant tumor-specific cell death *via* the intrinsic apoptosis pathway in CRC cells.

**Table 1:** IC_50_ of Cud C and 5-fluorouracil in colorectal cancer and non-transformed colon epithelial cells.

Colorectal Cancer Cells	IC_50_ Cud C (μM)	IC_50_ 5-fluorouracil (μM)
KM12	7.77 ± 1.33	26.27 ± 2.08
Caco-2	9.01 ± 2.38	35.0 ± 1.11
HT29	16.88 ± 0.65	35.47 ± 2.24
HCC2998	22.18 ± 5.54	43.72 ± 1.07
SW48	24.74 ± 2.34	24.42 ± 1.13
HCT116	34.67 ± 3.43	33.71 ± 1.06
CCD 841 CoN	>100	33.19 ± 2.25

### Microarray and *in silico* prediction identify PI3K-AKT as a target of Cud C

**Fig 2:**
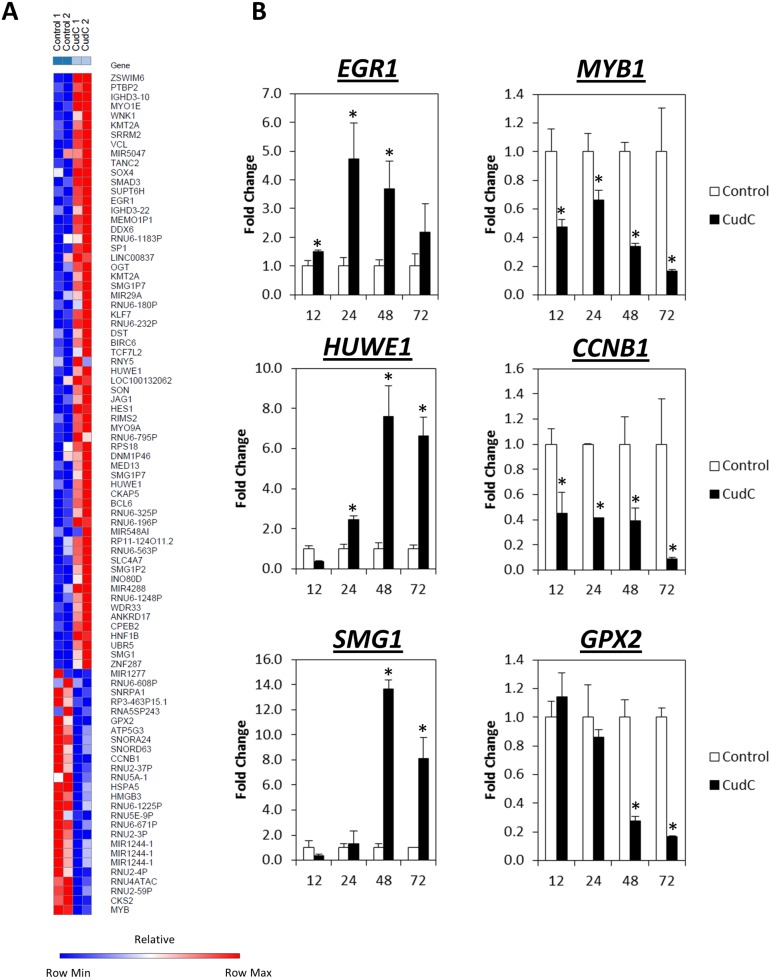
Differential gene expression regulated by cudraflavone C in Caco-2 cells. (A) Heatmaps generated based on the genes regulated by Cud C. Caco-2 cells were exposed to 10 μM Cud C for 48 hours. GeneChip^®^ Human Transcriptome Array 2.0 (Affymetrix, USA) was applied. Gene expression changes that ≥2-fold were considered significant. Control 1 and Control 2 represent gene expression from cells treated with vehicle control (1% DMSO); Cud C 1 and Cud C 2 were gene expression from cells treated with Cud C (10 μM). (B) qPCR was used to validate the microarray data. Caco-2 cells were exposed to 10 μM Cud C for 12, 24, 48 or 72 hours The left and right panels present genes that are up and down-regulated respectively. All data represent the mean ± s.d. from at least three independent experiments. Symbol “*” indicates the statistical significance concluded from Student’s independent *t*-test with p-value ≤0.05.

To determine the mechanism underlying the anti-tumor activities of Cud C, microarray gene profiling was performed on Caco-2 cells following treatment with 10 μM Cud C for 48 hours. Compared to the control cells, a total of 63 genes were up-regulated (fold change ≥2) while 26 genes were down-regulated as a result of Cud C exposure (Fig 2A).

We found that three of the most highly induced genes after Cud C exposure were *EGR1* (Early Response Growth-1), *HUWE1* (HECT, UBA and WWE domain containing 1) and *SMG1* (Suppressor of morphogenesis in genitalia-1) which have been reported to modulate apoptosis and cell cycle [34–36]. In contrast, Cud C downregulated *MYB1* (v-myb avian myeloblastosis viral oncogene homolog), *CCNB1* (cyclin B1) and *GPX2* (Glutathione peroxidase 2), which have been shown to promote cancer proliferation and metastasis [37–44]. We validated the aforementioned genes in response to Cud C by qPCR in samples extracted from Caco-2 and KM12 cells. Consistent with the microarray analyses, we observed time-dependent induction of *EGR1*, *HUWE1* and *SMG1*, and time-dependent suppression of *MYB1*, *CCNB1* and *GPX2*, following treatment with Cud C in both cell lines (Fig 2B and S2 Fig).

Next, we queried Cud C-response gene signature against the Connectivity Map (CMap) resource [26, 27]. Mining data generated from a reference collection of gene-expression profiles of cultured human cells treated with bioactive small molecules, using pattern matching software, the CMap can be used to find connections among small molecules sharing a mechanism of action. CMap analysis revealed seven small molecules connections to Cud C, all of which were either direct or indirect inhibitors PI3K-AKT signaling or protein synthesis (Table 2). These included Wortmannin and LY-294002 which are potent inhibitors of PI3K [45–47], and protein synthesis inhibitors puromycin and cycloheximide. We also identified thioridazine, an antipsychotic agent which was recently shown to inhibit the PI3K-AKT-mTOR pathway in endometrial or cervical cancer cells [48, 49]; perhexiline, a Ca^2+^ channel blocker that also targets PI3K-AKT-mTOR dependent autophagy [50]; and betulin, a plant-derived inhibitor of sterol regulatory element-binding proteins that has also been shown to inhibit PI3K-AKT-mediated growth of hepatoblastoma cells [51, 52].

**Table 2:** Top 20 pharmaceutical perturbagens exhibiting positive correlation to the gene signature induced by Cud C treatment.

Rank	Drug name	Score	P-value	Mechanism of Action
1	Puromycin	0.685	< 1.00E-06	Protein synthesis inhibitor
2	Wortmannin	0.581	< 1.00E-06	PI3K-AKT inhibitor
3	LY-294002	0.426	< 1.00E-06	PI3K-AKT inhibitor
4	Thioridazine	0.323	8.00E-05	Anti-psychotic drug
5	Perhexiline	0.620	1.00E-04	Increases myocardial efficiency
6	Camptothecin	-0.608	1.20E-04	Topoisomerase 1 inhibitor
7	Norethynodrel	-0.488	1.60E-04	Steroidal progestin
8	Hexetidine	0.518	1.80E-04	Anti-microbial
9	Emetine	0.495	4.00E-04	Anti-microbial
10	BCB000039	-0.561	5.20E-04	-
11	Nitrofurantoin	-0.357	5.80E-04	Anti-microbial
12	Lanatoside C	0.566	5.80E-04	Cardiac glycoside
13	Quinidine	-0.578	7.20E-04	Heart anti-arrhythmic
14	Helveticoside	0.499	1.13E-03	-
15	Nadolol	0.458	1.15E-03	Non-selective beta blocker
16	Methotrexate	-0.304	1.40E-03	Anti-metabolite and anti-folate
17	Podophyllotoxin	0.568	1.95E-03	Topoisomerase 2 inhibitor
18	Thonzonium bromide	0.570	2.05E-03	-
19	Cycloheximide	0.805	2.27E-03	Protein synthesis inhibitor
20	Betulin	0.517	2.56E-03	Sterol regulatory element-binding proteins inhibitor

Similarly, an *in silico* study using SwissTargetPrediction, was used to predict putative targets for Cud C. SwissTargetPrediction is a web-based cheminformatic tool designed to accurately predict targets of novel, uncharacterized bioactive molecules against a set of ≥ 300,000 known ligands. The results of SwissTargetPrediction also indicated that there is significant similarity between Cud C and AKT inhibitors (S3 Fig). Based on the data from the microarray, CMap and SwissTargetPrediction, we hypothesize that Cud C might exert its anti-tumor effects by regulating PI3K-AKT signaling.

### Cud C inhibits PI3K activities

**Fig 3:**
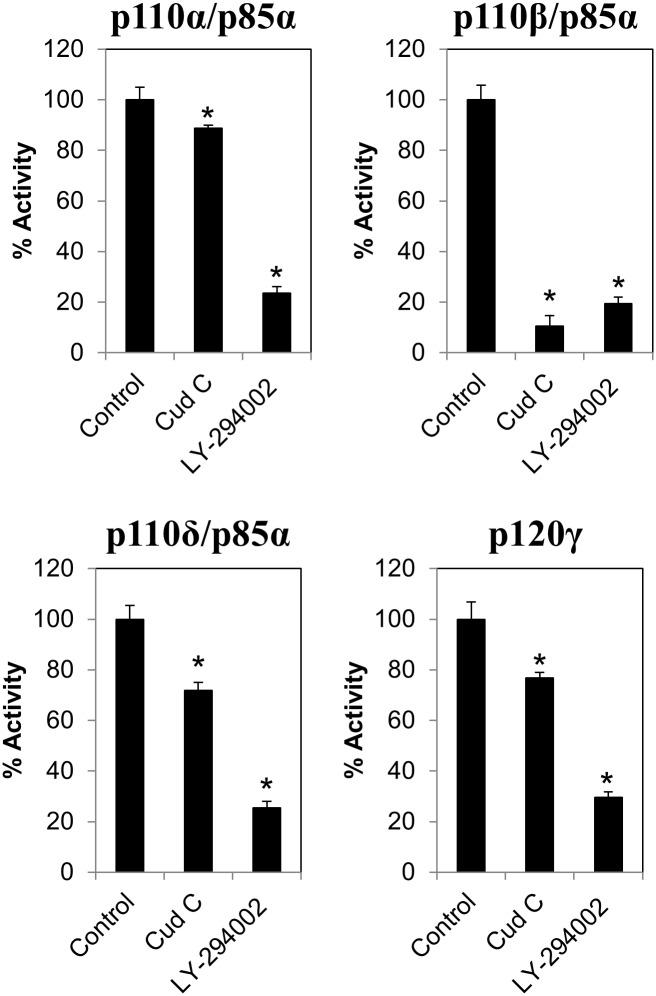
Cudraflavone C inhibits PI3K activity. The effect of negative control (1%DMSO), Cud C or LY-294002 (100 μM) on p110α/p85α, p110β/p85α, p110δ/p85α, and p120γ PI3K activity were quantified using the PI3K-Glo^™^ Class I Profiling Kit. All data represents the mean ± s.d. from at least three independent experiments. Symbol “*” presents the statistical significance concluded from Student’s independent *t*-test with p-value ≤0.05.

In order to confirm the effects of Cud C on PI3K activities, Cud C was incubated with p110α/p85α, p110β/p85α, p110δ/p85α, and p120γ PI3K in a cell free PI3K assay. As shown in Fig 3, Cud C significantly (*p* < 0.05) inhibited all PI3K activities. Interestingly, the inhibition by Cud C on p110β/p85α PI3K activity was as potent as the inhibition by LY-294002, a known PI3K inhibitor. Relatively, the inhibition was more selective towards p110β/p85α than other PI3K isoforms. The data suggest Cud C could be a selective p110β/p85α PI3K inhibitor.

### Cud C inhibits AKT phosphorylation in CRC

**Fig 4:**
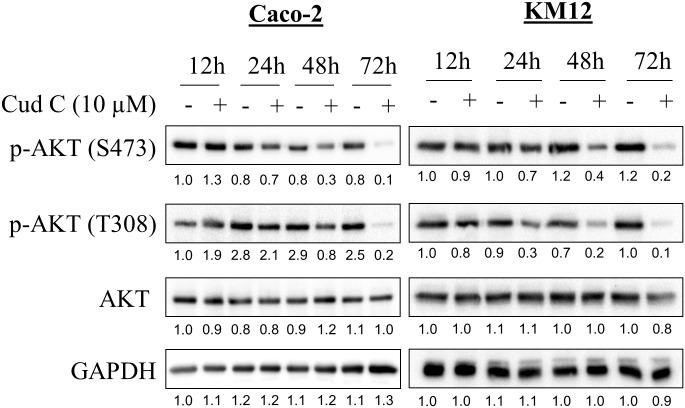
Cudraflavone C inhibits AKT phosphorylation in Caco-2 and KM12 cells. Caco-2 and KM12 cells were exposed to either DMSO (1%) or Cud C at 10 μM for 12, 24, 48 and 72hours. Protein lysates were subjected to SDS-PAGE. GAPDH was used as loading control. Numbers displayed below each blot denote the ratio of phosphorylated to total AKT.

To directly test whether Cud C modulated AKT signaling, we evaluated the levels of AKT phosphorylation in CRC cells following Cud C treatment. As highlighted in Fig 4, S473 and T308 AKT phosphorylation were both inhibited by Cud C in Caco-2 and KM12 cells at 24, 48 and 72 hours, while the total non-phosphorylated level of AKT remained unchanged. Of note, S473 and T308 AKT phosphorylation are required for its oncogenic function [53, 54]. Since PI3K-AKT signaling is often connected to PTEN and NFκB signaling [55], we also evaluated whether Cud C might induce PTEN or inhibit NFκB to induce tumor-specific cell death. Cud C affected neither PTEN expression nor NFκB activity, suggesting that Cud C inhibits PI3K-AKT signaling in CRC cells independently of PTEN and NFκB (S4 Fig). Of note, no PTEN expression was observed in KM12 cells, consistent with findings by Jhawer et al (2008) which reported that KM12 cells harbor a truncating mutation in PTEN [56].

### Activation of AKT abrogated apoptotic cell death induced by Cud C

**Fig 5:**
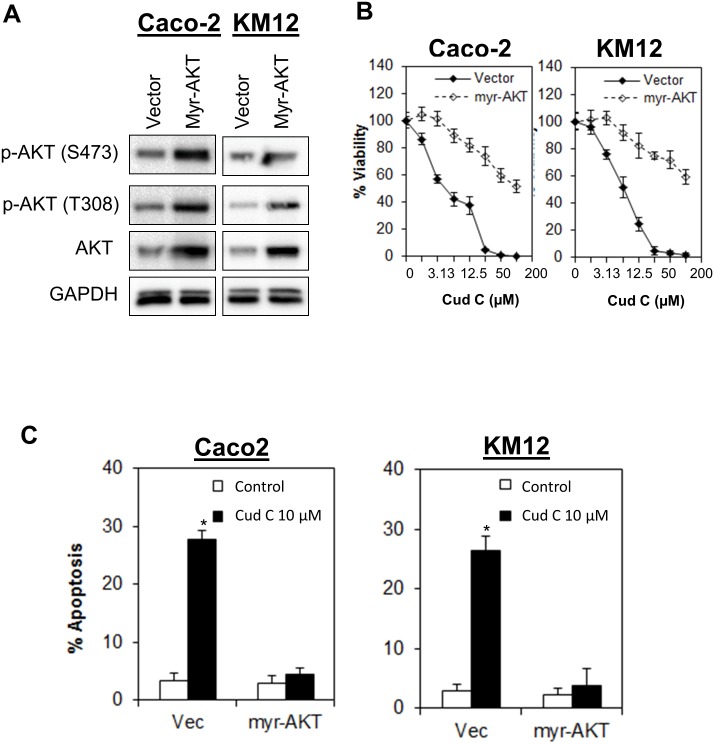
Ectopic expression of myr-AKT confers resistance to cudraflavone C. (A) Caco2 and KM12 cells were transfected with myristoylated AKT for 24 hours before treatment of cells with test agent. Lysates were collected at 48 hours after transfection for immunoblotting analysis. (B) Cell proliferation was quantified by CellTiterGlo^®^ and (C) Apoptotic cell death was quantified by annexin V/7-AAD flow cytometry. All data represent the mean ± s.d. from at least three independent experiments. Symbol “*” presents the statistical significance concluded from Student’s independent *t*-test with p-value ≤0.05.

To further confirm that the anti-tumor potential of Cud C is driven by PI3K-AKT signaling, we overexpressed the constitutively active myristoylated AKT in CRC cells. The activation completely abrogated induction of apoptosis and significantly reduced the sensitivity of Caco-2 and KM12 towards Cud C (Fig 5). Together, these finding demonstrate that the anti-tumor activity of Cud C in CRC cells is mediated through inhibition of PI3K-AKT signaling.

## Discussion

Cudraflavone is a prenylflavone originally isolated from the root of *Cudrania tricuspidata* (Carr.) Bur. (Moraceae) [19]. Interestingly, *Cudrania tricuspidata* (Carr.) Bur is applied as a folk remedy in Korea [57], and has demonstrated anti-inflammatory activities [58]. To date, a total of 3 analogs of Cud have been isolated, including Cud A, B and C [19, 59]. Cud A has been shown to inhibit melanin production through suppression of tyrosinase [60] and potentially evokes antidepressant activities *via* inhibition of brain monoamine oxidase [61]. Similarly, Cud B also inhibited melanin production [62] and brain monoamine oxidase [61]. In addition, Cud B inhibited cancer cells growth and induced mitochondrial-dependent apoptosis in human oral cancer cells through regulation of MAPK, NFκB, and SIRT1 signaling [18]. While several benefits of Cud C have been reported recently, such as inhibition of melanin production [13], inhibition of pancreatic lipases [14], and HSV activation [15], few studies have been conducted to evaluate its anti-proliferative activities [16, 17]. Virtually nothing is known regarding the mechanism underlying the antitumor activities of Cud C.

Here, we discover that Cud C selectively inhibit the growth of CRC (KM12, HT29, Caco2, SW48, and HCT116) but not in the normal isogenic colon cells (CCD 841 CoN). This result demonstrates the selective antitumor effect of Cud C in cancer cells by sparing the non-cancer cells. In addition to that, Cud C induces mitochondrial-dependent apoptosis, corroborated by mitochondrial membrane depolarization through JC-1 staining and induction of caspase 3/7 and 9 activities in CRC cells, but not in normal colorectal epithelial cells. Gene expression analyses showed that Cud C exhibited a gene profile that was similar to PI3K-AKT inhibitors (Wortmannin and LY-294002) suggesting that Cud C may induce its anti-tumor effects through the regulation of PI3K-AKT independent of NFκB signaling. Indeed, treatment of CRC cells with Cud C induced significant reduction in AKT phosphorylation in 2 independent CRC cells (KM12 and Caco-2), while the ectopic expression of constitutively active myristoylated AKT completely abrogated the antitumor effects of Cud C. These results suggest that the antitumor effect of Cud C is PI3K-AKT dependent.

PI3Ks are lipid kinases that phosphorylate the 3’-hydroxyl of phosphatidylinositol and phosphoinositides. Upon activation, PI3K is recruited to the plasma membrane and converts phosphatidylinositol-4,5-bisphosphate (PIP_2_) to phosphatidylinositol-3,4,5-trisphosphate (PIP_3_) [63]. The resulting PIP_3_ in turn creates binding sites for specific, lipid-binding domains on many intracellular signaling proteins, including phosphoinositide-dependent kinase (PDK)-1 and the serine-threonine kinase AKT. The activation of AKT is triggered by the phosphorylation of AKT at Thr308 and Ser473. Following activation, AKT translocates to the cytoplasm and nucleus, where it phosphorylates effector proteins to regulate cell survival, protein synthesis, proliferation, and metabolism [64]. Thus, PI3K-AKT pathway mediates a multitude of oncogenic signals which include cell cycle, apoptosis, protein synthesis, cell growth and proliferation. Aberrations of this gene may therefore lead to cancer formation, as AKT is at the crossroads of many tumour suppressor and oncogenic signalling pathways. Indeed, mutations of the components of the PI3K-AKT signaling have been studied to result in oncogenesis. Anomalies of this pathway, such as overexpression of p-AKT, is widely implicated to have a role in carcinogenesis or cancer cell survival in numerous cancers, including that of cancers [65–72]. The mutant p53-R273H in cancer cells may mediate cancer cell survival and anoikis resistance by activating AKT and suppressing BCL-2-modifying factor [22]. Activation of AKT in CRC patients were found to have poorer prognosis and survival [73, 74].

Recent genomic analyses of human CRC indicate that more than 30 disrupted pathways are related to PI3K signaling [75]. It is no surprise that many inhibitors of the PI3K-AKT-mTOR signaling pathway have been developed and have demonstrated efficacy in inhibiting CRC proliferation in preclinical studies [63, 76–78]. However, many of these agents, such as rapamycin and its analogs, are predominantly mTOR inhibitors. In contrast, PI3K and AKT inhibitors are still under development, with none thus far reaching the bedside [79]. First-generation PI3K inhibitors Wortmannin and LY294002, while effective in pre-clinical models, were limited by poor pharmacokinetic properties [80]. Similarly, few AKT-targeting agents have been developed, examples of which include miltefosine, which has completed a phase III clinical testing [81]; and a glycoside (PBI-05204) that was first isolated from *Nerium oleander* [82]. The combination of capecitabine (Xeloda, Roche) and perifosine (KRX-0401, Aeterna Zentaris/Keryx), an orally active PI3K-AKT inhibitor, has showed good clinical outcomes in patients with CRC[83, 84]. Other agents that still undergoing clinical trials are GSK-2141795 (Glaxo-SmithKline), allosteric inhibitor such as MK2206 (Merck) and catalytic inhibitors such as GSK-690693 (Glaxo-SmithKline) in various solid tumors and lymphoma [83, 85]. To date, we are assured that PI3K-AKT inhibitors have reasonable efficacy and favorable safety profile. However, there are many unresolved issues with each clinical trial agent that hinder the progress of these agents to bedside[85]. The need for a better molecule targeting this pathway is still in great demand.

In view of the challenges in developing PI3K-AKT inhibitors, current drug development efforts are moving towards developing isoform specific PI3K inhibitors especially p110β PI3K inhibitors, mindful of PI3K-independent roles of p110β in tumorigenesis of tumors possessing Phosphatase and Tensin (PTEN) loss of functions [86]. PTEN is a well-established tumor suppressor [87]. Low PTEN expression is related to CRC-liver metastasis, resistance to targeted chemotherapy agent such as cetuximab, and thus poorer prognosis [88, 89]. In this study, we confirmed Cud C is a selective p110β PI3K inhibitor. However, our results concluded Cud C did not induce or inhibit PTEN expression in Caco2 and KM12 cells. Jing Ni and coworkers illustrated the potential of KIN-193, a p110β PI3K inhibitor with potential anti-tumor effect in breast and prostate xenograft models [90]. Shuttleworth and co-workers developed a selective dual p110β/δ inhibitor, KA2237, which is currently under Phase I clinical evaluation to target hematological cancer [91]. Scant literature is available pertaining to the development of selective p110β PI3K inhibitors. Therefore, in this study, Cud C, a potent selective p110β PI3K-AKT inhibitor independent of PTEN and NFκB with selective anti-tumor activities against CRC represents a promising developmental anticancer candidate. Cud C may open up a new area of study and drug design approaches, leading to the design of more effective and selective p110β PI3K-AKT inhibitor which are independent of PTEN and NFκB.

## Conclusions

Our study demonstrates that Cud C is a PI3K-AKT inhibitor with selective anti-tumor activities against CRC cells. These results imply that Cud C offers tremendous potential for further development as a therapeutic agent for CRC.

## Supporting Information

S1 Fig Confirmation of Cud C’s structure using ^1^H and ^13^C NMR of Cud C. (TIF)

S2 Fig Validation of microarray data. KM12 cells were exposed to 10 μM Cud C for 12, 24, 48 or 72 hours and followed by qPCR. The left and right panels depict genes that are up-regulated and down-respectively. All data represents the mean ± s.d. from at least three independent experiments. Symbol “*” presents the statistical significance concluded from Student’s independent *t*-test with p-value < 0.05.
(TIF)

S3 Fig Predicted targets of Cud C as determined by SwissTarget Prediction. Scores were used to rank the targets. The distribution of targets was concluded in a pie chart.
(TIF)

S4 Fig Effects of Cud C on PTEN expression and NFκB activity. (A) Caco2 and KM12 cells were exposed to 10μM Cud C for 48 and 72 hours and protein lysates were harvested for PTEN immunoblotting. (B) NFκB reporter cells were treated with 0.1% DMSO, Cud C (1, 10 or 100μM) or TNFα (100ng/mL) for 48 hours. The relative NFκB activity of Cud C or TNFα was calculated as a ratio of normalized activity in Cud-C treated cells to normalized activity in cells treated with 0.1% DMSO. All data represents the mean ± s.d. from at least three independent experiments. Symbol “*” presents the statistical significance concluded from Student’s independent *t*-test with p-value < 0.05.
(TIF)

S1 Table Forward and reverse primer sequences for quantitative RT-PCR. (DOCX)
