# Supplementary figures and images for: Cudraflavone C Induces Tumor-Specific Apoptosis in Colorectal Cancer Cells through Inhibition of the Phosphoinositide 3-Kinase (PI3K)-AKT Pathway
Source: PLoS One. 2017 Jan 20;12(1):e0170551. doi: 10.1371/journal.pone.0170551 (PMC5249192; doi:10.1371/journal.pone.0170551)

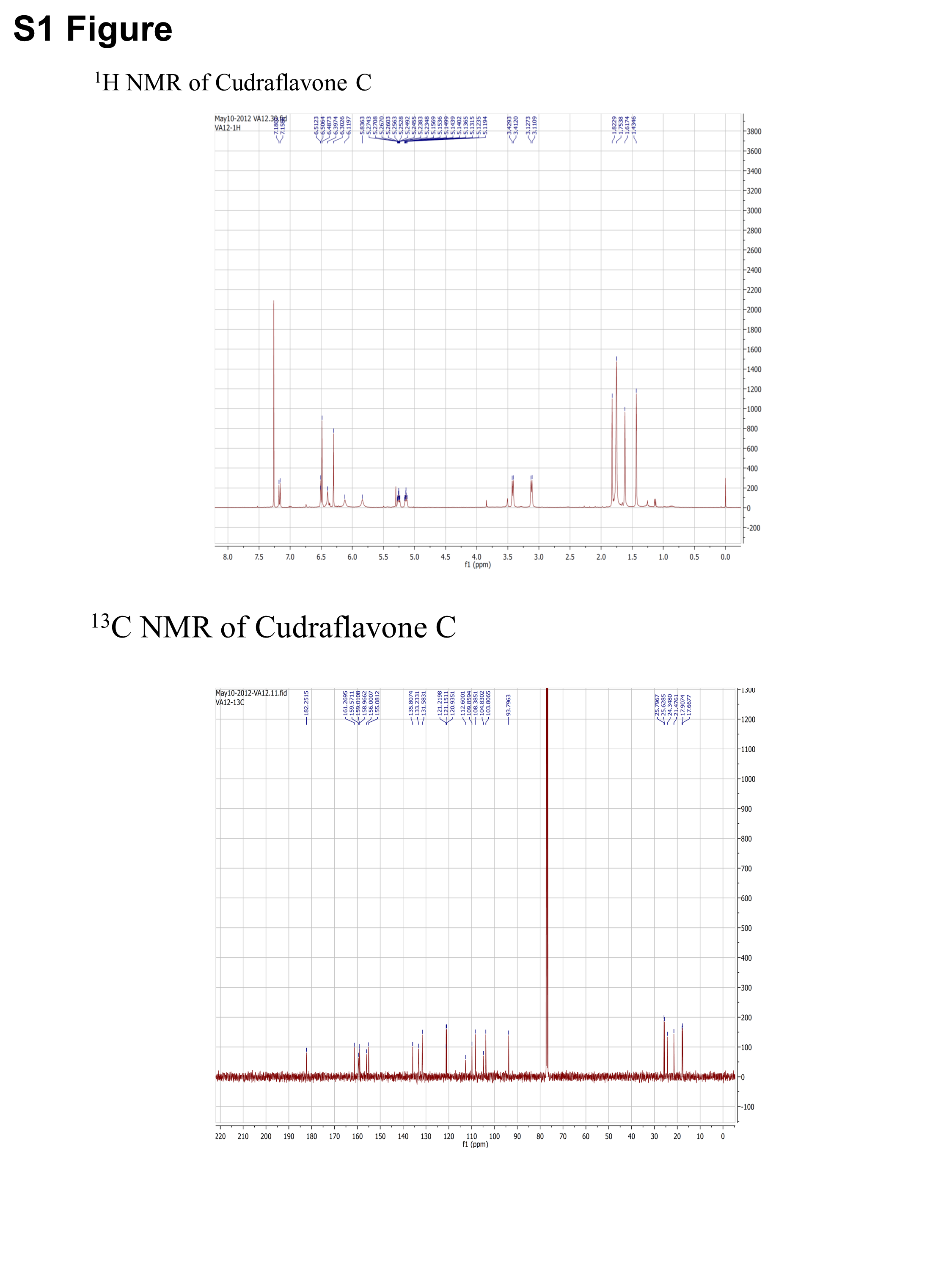

Supplement: S1 Fig — (TIF) [file pone.0170551.s001.TIF]

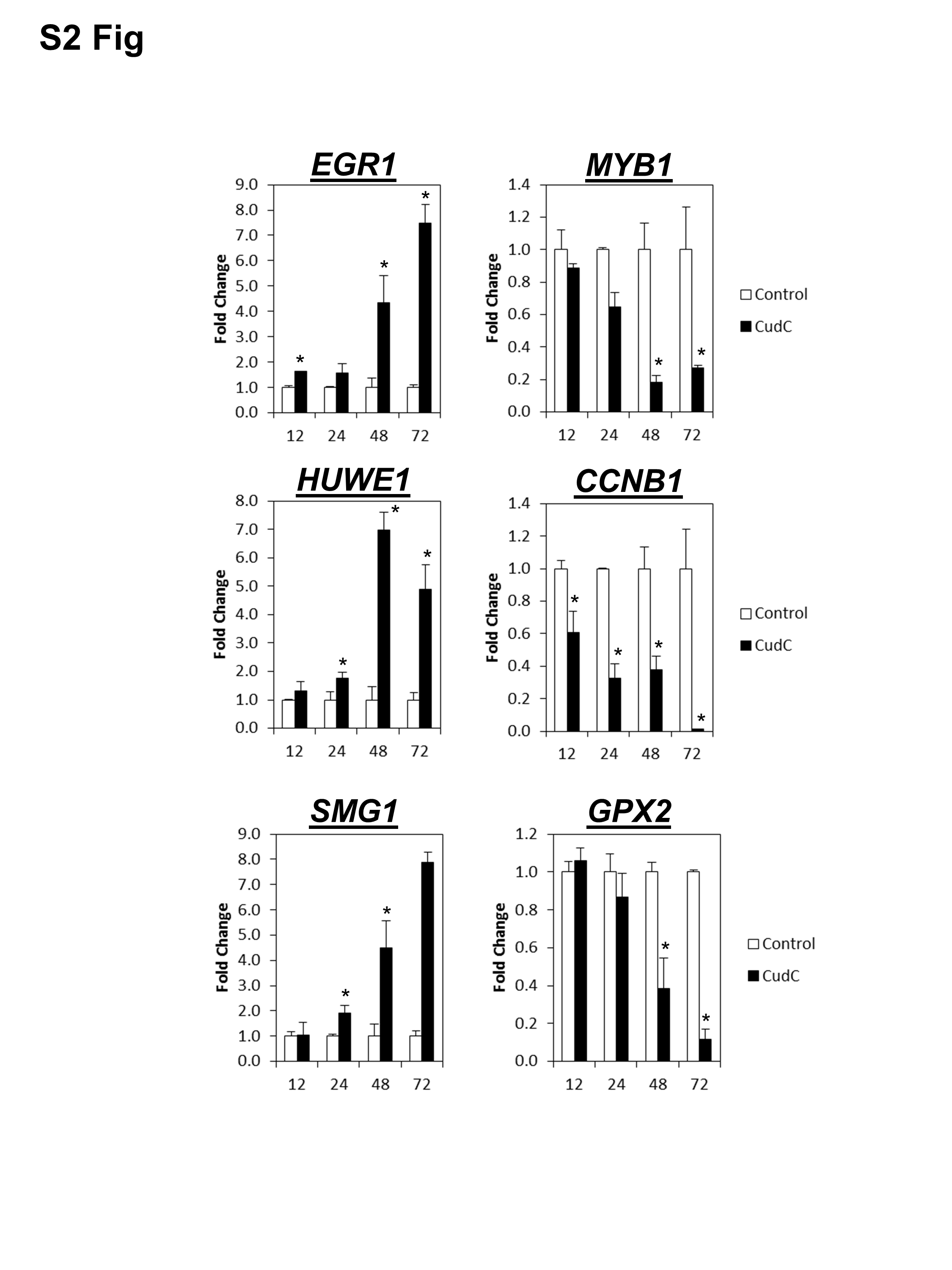

Supplement: S2 Fig — KM12 cells were exposed to 10 μM Cud C for 12, 24, 48 or 72 hours and followed by qPCR. The left and right panels depict genes that are up-regulated and down-respectively. All data represents the mean ± s.d. from at least three independent experiments. Symbol “*” presents the statistical significance concluded from Student’s independent t-test with p-value < 0.05. (TIF) [file pone.0170551.s002.TIF]

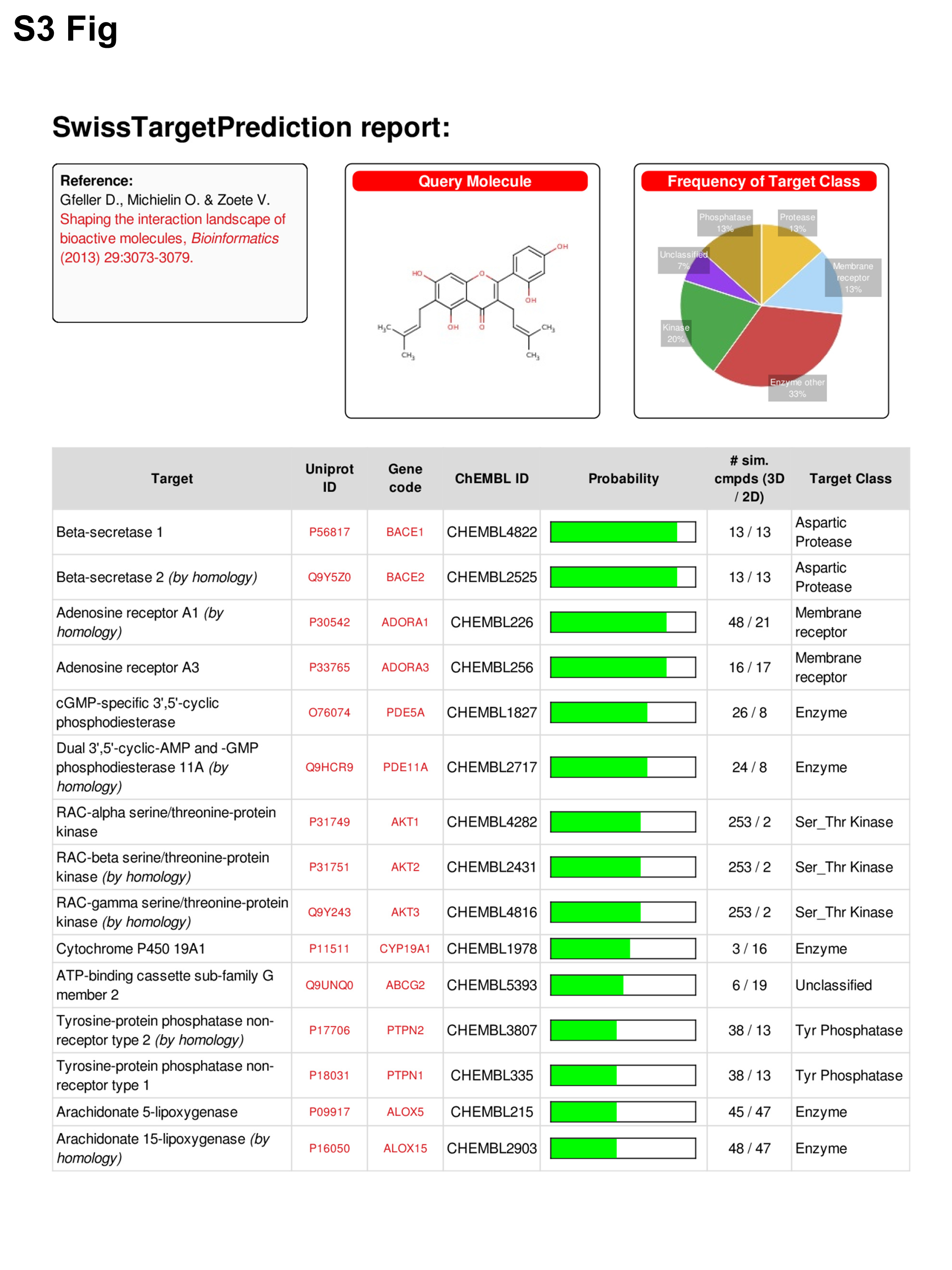

Supplement: S3 Fig — Scores were used to rank the targets. The distribution of targets was concluded in a pie chart. (TIF) [file pone.0170551.s003.TIF]

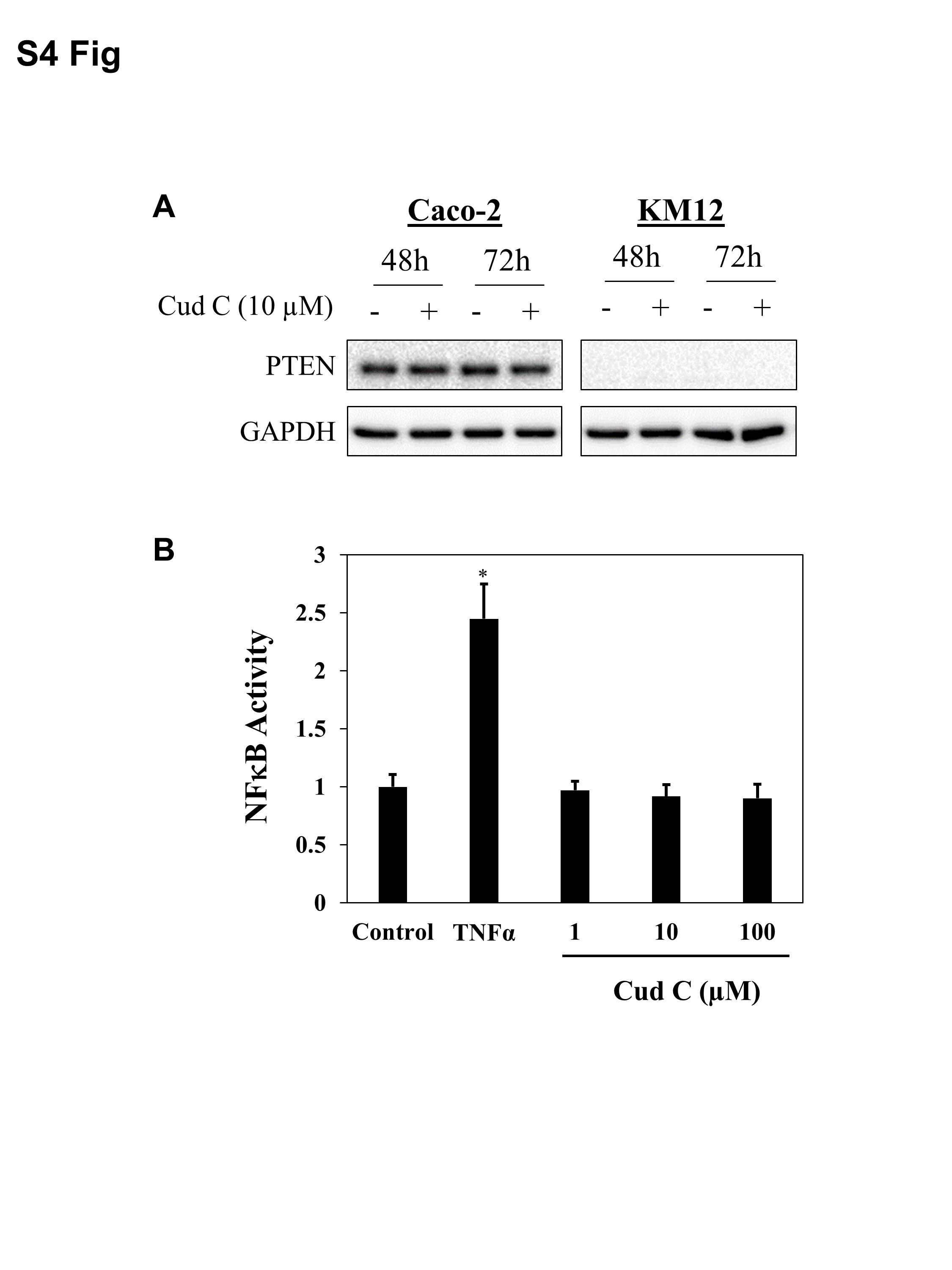

Supplement: S4 Fig — (A) Caco2 and KM12 cells were exposed to 10μM Cud C for 48 and 72 hours and protein lysates were harvested for PTEN immunoblotting. (B) NFκB reporter cells were treated with 0.1% DMSO, Cud C (1, 10 or 100μM) or TNFα (100ng/mL) for 48 hours. The relative NFκB activity of Cud C or TNFα was calculated as a ratio of normalized activity in Cud-C treated cells to normalized activity in cells treated with 0.1% DMSO. All data represents the mean ± s.d. from at least three independent experiments. Symbol “*” presents the statistical significance concluded from Student’s independent t-test with p-value < 0.05. (TIF) [file pone.0170551.s004.TIF]
