# Supplementary material for: Cudraflavone C Induces Tumor-Specific Apoptosis in Colorectal Cancer Cells through Inhibition of the Phosphoinositide 3-Kinase (PI3K)-AKT Pathway
Source: PLoS One. 2017 Jan 20;12(1):e0170551. doi: 10.1371/journal.pone.0170551 (PMC5249192; doi:10.1371/journal.pone.0170551)
Supplement: S1 Table — (DOCX) [file pone.0170551.s005.docx]

**Supplemental Table 1: IC_50_ of 5-fluorouracil in colorectal cancer cell lines**

| **Colorectal cancer cell lines** | **IC_50_ (µM)** |
| --- | --- |
| KM12 | 26.27 ± 2.08 |
| Caco-2 | 35.0 ± 1.11 |
| HT29 | 35.47 ± 2.24 |
| HCC2998 | 43.72 ± 1.07 |
| SW48 | 24.42 ± 1.13 |
| HCT116 | 33.71 ± 1.06 |
